# DNA-dependent protein kinase catalytic subunit (DNA-PKcs) contributes to incorporation of histone variant H2A.Z into nucleosomes

**DOI:** 10.1007/s13238-019-0632-1

**Published:** 2019-05-20

**Authors:** Ling-Yao Wang, Yun-xiao He, Min Li, Jian Ding, Yi Sui, Joan W. Conaway, Ronald C. Conaway, Fei Wang, Jingji Jin, Yong Cai

**Affiliations:** 1 School of Life Sciences, Jilin University, Changchun, 130012 China; 2 Stowers Institute for Medical Research, 1000E 50th Street, Kansas City, MO 64110 USA; 3 National Engineering Laboratory for AIDS Vaccine, Jilin University, Changchun, 130012 China; 4 Key Laboratory for Molecular Enzymology and Engineering, The Ministry of Education, Jilin University, Changchun, 130012 China

**Dear Editor,**

Current research has demonstrated that the dynamic distribution of histone variant H2A.Z at specific loci can be regulated by ATP-dependent chromatin remodelers, histone chaperones and histone post-translational modifications (PTMs). ATP-dependent chromatin remodelers such as the yeast Swr1, p400/Tip60 and human SRCAP complexes are mainly responsible for the presence of H2A.Z at specific loci by replacement of H2A with H2A.Z (Ruhl et al., 2006). On the other hand, it is widely believed that the H2A.Z substituted into nucleosomes removed by the INO80 chromatin remodeling complex (Alatwi and Downs, 2015). It has also been shown that the INO80 complex plays a role in removing H2A.Z at the p53-binding site of *p21* in response to doxorubicin in U2OS osteosarcoma cells (Ding et al., 2018). In addition to the chromatin remodeler, ANP32E is an H2A.Z chaperone that specifically removes H2A.Z from the nucleosomes (Obri et al., 2014). When DNA damage occurs, H2A.Z is transiently retained at double strand breaks (DSBs); this accumulation can be removed by ANP32E via direct interaction with the αC-helix of H2A.Z (Mao et al., 2014).

Recent research indicates that ANP32E stabilizes H2A.Z by inhibiting protein phosphatase 2A (PP2A). Inhibition of PP2A by ANP32E prevents nuclear exclusion and dephosphorylation of H2A.Z at serine 9 (Shin et al., 2018), suggesting that the phosphorylation of H2A.Z may be involved in its distribution at specific loci. In plants, phosphorylation of H2A at serine 95 has been implicated in the regulation of flowering time and deposition of H2A.Z onto nucleosomes (Su et al., 2017), suggesting that the regulation of H2A.Z distribution at DSBs may be more complicated than has been presumed, and may require the coordination of multiple regulatory mechanisms.

DNA-dependent protein kinase (DNA-PK) is the largest protein kinase in the phosphoinositide-3-kinase-related kinase (PIKK) family, and comprises a DNA-dependent protein kinase catalytic subunit (DNA-PKcs) and Ku70/80 heterodimer (Davis et al., 2014). In U2OS cells, ionizing radiation (IR) induces DNA-PK-dependent phosphorylation of nuclear fumarase (FH) at threonine 236, resulting in the binding of FH to H2A.Z at DSBs, and further promoting accumulation of the DNA-PK complex at DSB sites and initiating the non-homologous end-joining (NHEJ) repair pathway. These results suggest a possible relationship between DNA-PK and H2A.Z during DNA damage.

In this report, we present evidence for the first time that DNA-PKcs-containing components purified through multi-chromatography purification steps from HeLa nuclear extracts can incorporate H2A.Z-H2B dimers into reconstituted nucleosomes. To identify a novel human H2A-H2A.Z exchange-enzyme, protein purification was initiated with HeLa S3 nuclear extracts prepared from 5 × 10^10^ cells. Figure 1A presents a schematic of the protein purification and enzyme activity tracking process. Then, using established *in vitro* H2A.Z deposition assay (Figs. 1B and S1), we tracked the chromatographic column fractions with H2A.Z deposition activity. H2A.Z substituted into reconstituted nucleosomes was evaluated by western blot with anti-Flag antibody (anti-H3 or CBB-stained protein gel was as nucleosomes loading control). At first, active components from the phosphocellulose chromatography (P11, fraction size: 10 mL) fractions (fractions 4–6) were found in eluted stepwise with 0.3 mol/L NaCl (Fig. 1C). Mixed 4–6 fractions were applied to the TSK gel DEAE-5PW HPLC ion-exchange column, and fractions 11 and 12 exhibited enzyme activity (Fig. 1D). However, the activity was much higher in fraction 11 than in 12. Therefore, fraction 11 was chosen for the subsequent purification process. H2A-H2A.Z-exchange enzyme-restricted components were found in fractions 26 and 27 of the TSK SP-5PW column (Fig. 1E). The pooled sample (SP-5PW fractions 26 + 27, SP mix) was used for enzyme activity confirmation experiments. To confirm that the enzyme activity was derived from the protein component, the SP mix was heated at 95 °C for 10 min before being applied to the *in vitro* H2A-H2A.Z-exchange assay. The heated sample had complete loss of enzyme activity (lane 3), suggesting that the enzyme activity was in the protein component (Fig. S2). In addition, the enzyme activity was increased in an ATP-dependent manner (Fig. 1F, lanes 3–5) compared to reactions without ATP (lane 2). And this enzyme activity was decreased by addition of ATPγS (Fig. 1G, lanes 3–5 compared to lane 2). To further purify and identify this enzymatically active component, the SP mix was subjected to a 10%–40% glycerol gradient (Fig. 1A). Detection of enzyme activity suggested that the activity was mainly in fractions 18–20 (Fig. 1I). The polypeptides in these fractions were visualized by silver staining (Fig. 1H), and the pooled fractions 17–21 underwent mass spectrometry. The results showed that the main component of the pooled sample was DNA-PKcs, and the protein coverage were 42.5% (Table S1), indicating that the DNA-PKcs containing component may catalyze the H2A.Z replacement process.

**Figure 1:**
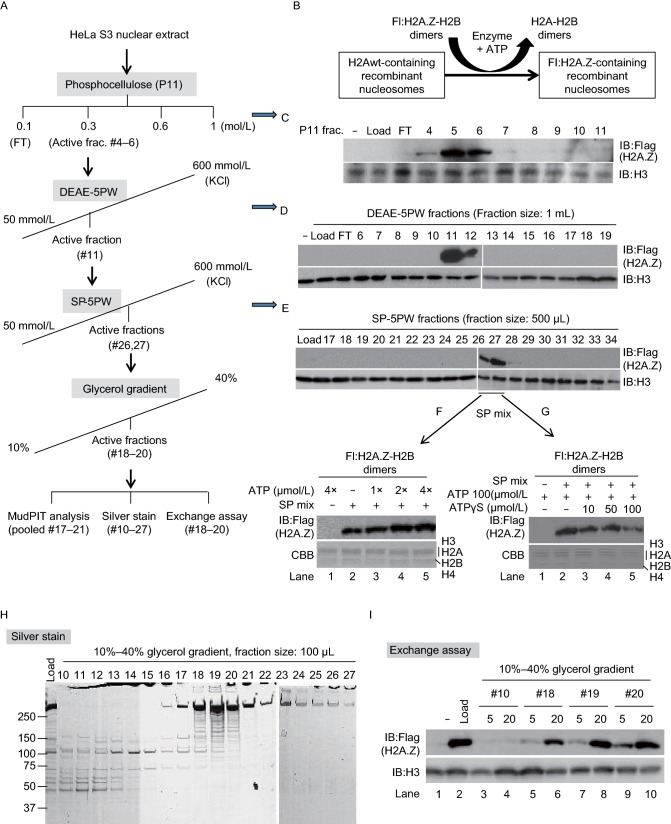
**Fractions with H2A.Z-H2A replacement activity were purified by multiple chromatography purification steps**. (A) Schema of purification steps. (B) Enzyme activity reaction diagram. (C–E) Tracking for components with H2A.Z-H2A replacement activity. (F–G) SP mix exhibited ATP-dependent enzyme activity. (H) Silver stained polypeptides of 10%–40% glycerol gradient chromatography fractions. (I) Enzymatically active fractions of 10%–40% glycerol gradient

To further confirm that the DNA-PKcs-containing component does participate in catalyzing the H2A.Z deposition process, HeLa nuclear extracts from 5 × 10^10^ cells were used for a second round of protein isolation, and active fractions were purified by tracking H2A.Z deposition enzyme activity with an *in vitro* assay system. Fig. 2A presents a schematic of the protein purification. Active fractions 5–7 from stepwise eluates of P11 were applied to the TSK SW4000 hydrophilic chromatography column. The DNA-PKcs protein in the SW4000 fractions was analyzed by Western blotting with specific antibody (Fig. 2B). The main peak of DNA-PKcs appeared in fractions 35–37. As expected, the enzyme activity was consistent with the DNA-PKcs peaks (fractions 35–37; Fig. 2C), suggesting that the DNA-PKcs-containing components possessed the catalytic H2A.Z replacement enzyme activity. In order to further confirm this hypothesis, we performed immunoprecipitation (IP) experiments in the fraction #37 using DNA-PKcs specific antibody. Then the supernatant after removing DNA-PKcs was applied to *in vitro* H2A.Z deposition assay. As expected, the enzyme activity was no longer shown after removal of DNA-PKcs (Fig. 2D, lanes 3–4 compared to lane 2). *In vitro* H2A.Z-deposition assay confirmed again that DNA-PKcs containing component (fraction #35) can incorporate H2A.Z into reconstituted nucleosomes in an ATP-dependent manner (Fig. 2E, Flag-H2A.Z/lanes 3–5), and this activity can be inhibited by ATPγS (Flag-H2A.Z/lane 6). Simultaneously, the endogenous H2A in nucleosomes was dose-dependently decreased (Fig. 2E, IB:H2A/lanes 2–5), suggesting that an exchange between H2A and H2A.Z occurred. To further confirm whether the enzyme activity was specific, we carried out a reverse exchange experiment using H2A.Z-containing nucleosomes and Flag-H2A-H2B dimer. As shown in Fig. 2F, although the DNA-PKcs containing component appeared to have weaker activity (lanes 3–5), the enzymatic activity was negligible compared to the Arp5-complex (lane 6). Arp5 (a subunit of INO80 complex) complex was purified from stably expressing Flag-Arp5 293FRT cells, indicating that the DNA-PKcs containing component is mainly responsible for incorporating H2A.Z into reconstituted nucleosomes.

**Figure 2:**
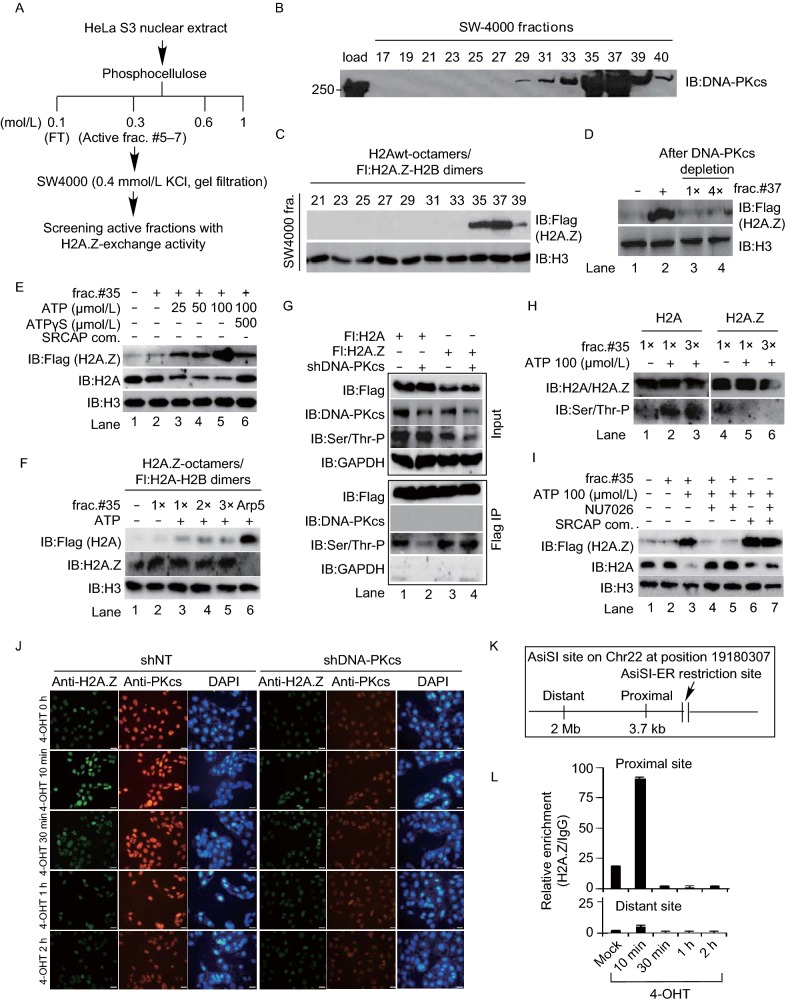
**DNA-PKcs-containing components possessed H2A.Z-H2A replacement enzyme activity**. (A) Schema of purification steps. (B) DNA-PKcs protein peak in the SW4000 purification fractions. (C) Enzyme activity is consistent with the protein peak of DNA-PKcs. (D) Lost enzyme activity after depletion of DNA-PKcs with specific antibody. (E) DNA-PKcs containing fraction (#35) exhibited ATP-dependent enzyme activity. (F) DNA-PKcs containing fraction was mainly responsible for depositing H2A.Z into reconstituted nucleosomes. (G) shDNA-PKcs-mediated reduction of H2A-phosphorylation. (H) Phosphor-modification of H2A by DNA-PKcs-containing component in *in vitro* assay. (I) DNA and kinase activity-dependent enzyme activity. (J) Immunofluorescence staining to detect H2A.Z (green) and DNA-PKcs (red) dynamic change in *Asi*SI-ER U2OS cells. DAPI staining shows total nuclei. Scale bar indicates 20 µm. (K) Primers for ChIP-qPCR at proximal and distal to *Asi*SI site on Chr22 position 19180307. (L) Accumulation of H2A.Z at 10 min was observed at proximal to *Asi*SI site after 4-OHT treatment

Nucleosomal DNA accessibility is modulated by charge-altering PTMs including acetylation, phosphorylation and methylation in the histone core (Hu et al., 2013). Although it has not been reported if the phosphorylation in histone core affects the deposition and removal of H2A.Z, but several studies have suggested that phosphor modification may also be associated with the distribution of H2A.Z at specific loci (Su et al., 2017; Shin et al., 2018). In our experimental conditions, reduced phosphor-H2A was observed in lentiviral-mediated DNA-PKcs knockdown cells (Fig. 2G, Flag IP/lane 2 compared to lane 1). This result was supported by *in vitro* phosphor-assay using *E*. *coli* expressed and purified H2A in the presence of SW4000 fraction #35. As shown in Fig. 2H, ATP-dependent phosphorylation only occurred on recombinant H2A (lanes 2–3 compared to lane 1), but not on H2A.Z (lanes 5–6 compared to lane 4). To further confirm that the H2A.Z deposition activity of DNA-PKcs-containing component may be related to its phosphorylating histone core, we performed the experiment shown in Fig. 2I. It was clear that DNA-PKcs, but not SRCAP, no longer had H2A.Z-deposition activity in the presence of DNA-PKcs inhibitor NU7026 (lanes 4–5 compared to lane 3), suggesting that DNA-PKcs-mediated histone phosphorylation may be involved in H2A.Z-deposition activity. These results indicated that the phosphor modification of the histone core by DNA-PKcs may relax the interaction between DNA and histones, thereby increasing the DNA accessibility of histone H2A.Z-H2B dimer and leading to an exchange between H2A.Z and H2A.

The highly conserved histone variant H2A.Z is frequently enriched at actively transcribed promoters, at DSBs, and in segregating chromosomes (Papamichos-Chronakis et al., 2011; Shin et al., 2018). To observe the dynamic distribution of H2A.Z during DNA damage, stably expressing *Asi*SI-ER U2OS cells were used (Qi et al., 2015) in subsequent experiments. The fused *Asi*SI contains a modified oestrogen receptor hormone-binding domain (ER) that only binds to 4-hydroxytamoxifen (4-OHT), and 4-OHT treatment induces nuclear localization of *Asi*SI-ER and generates DSBs (Littlewood et al., 1995). In our experimental conditions, the foci of H2A.Z at DSBs after 4-OHT treatment in stably expressing *Asi*SI-ER U2OS cells were only retained for a very short time. There was a short-lived peak at 10 min that quickly disappeared. However, this phenomenon was not seen in DNA-PKcs knockdown cells (Fig. 2J). To determine whether H2A.Z can be restricted to DSB sites, a chromatin immunoprecipitation (ChIP) assay on chromosome 22 was performed using H2A.Z-specific antibody in the *Asi*SI DSB induction system. Two primer sets on chromosome 22 used for amplifying ChIP DNA (Fig. 2K). As expected, the accumulation of H2A.Z on chromatin proximal to DSBs was only observed at 10 min (Fig. 2L). This transiently retained H2A.Z may be rapidly removed by INO80 and recruit DNA damage machinery (Lademann et al., 2017). Although there appears to be crosstalk among DNA-PKcs, histone phosphorylation, and H2A.Z; however, the underlying regulatory mechanisms are still unclear.

In summary, the regulatory mechanisms of H2A.Z distribution at specific loci are coordinated by multiple factors. Depending on the environment and stimuli to which cells are exposed, the enzymes and proteins that participate in H2A.Z redistribution are different. DNA-PKcs containing component as a potential novel H2A.Z regulatory factor may provide new insights into the functional mechanisms of intracellular H2A.Z.

## FOOTNOTES

This work was supported by National Natural Science Foundation of China (Nos. 31371311 and 31771421).

Jingji Jin, Yong Cai, JW Conaway and RC Conaway designed the research; Ling-Yao Wang, Yun-xiao He, Min Li, Jian Ding, Yi Sui, Fei Wang, Jingji Jin and Yong Cai conducted the research; Ling-Yao Wang and Jingji Jin analyzed data; and Ling-Yao Wang, Yong Cai and Jingji Jin wrote the manuscript.

Ling-Yao Wang, Yun-xiao He, Min Li, Jian Ding, Yi Sui, JW Conaway, RC Conaway, Fei Wang, Jingji Jin and Yong Cai declare that they have no conflict of interest.

This article does not contain any studies with human or animal subjects performed by the any of the authors.

## Electronic supplementary material

Below is the link to the electronic supplementary material.

Supplementary material 1 (DOCX 5027 kb)
