# Supplementary material for: DNA-dependent protein kinase catalytic subunit (DNA-PKcs) contributes to incorporation of histone variant H2A.Z into nucleosomes
Source: Protein Cell. 2019 May 20;10(9):694–9. doi: 10.1007/s13238-019-0632-1 (PMC6711948; doi:10.1007/s13238-019-0632-1)
Supplement: Supplementary file 1 — Supplementary material 1 (DOCX 5027 kb) [file 13238_2019_632_MOESM1_ESM.docx]

**Materials and Methods**

**Antibodies and chemicals:** Anti-H2A.Z (GTX108273) and anti-DNA-PKcs (GTX49138) was from GeneTex (Irvine, CA, USA). Anti-H3 (RLM3038) was obtained from Ruiying Biological (Suzhou, China). Anti-Flag (M2) (F3165) monoclonal antibody was from Sigma (St. Louis, MO, USA). Anti-DNA-PKcs (residues 3965–4127) and anti-GAPDH rabbit polyclonal antibodies were raised against bacterially expressed proteins (Jilin University, Jilin, China).

**Generation and growth of mammalian cell lines:** HEK293T, HeLa and *Asi*SI-ER-U2OS cells were maintained in Dulbecco’s Modified Eagle’s Medium (DMEM; Gibco Life Technologies, Gaithersburg, MD, USA) containing 10% fetal bovine serum (KangYuan Biology, Tianjin, China) and 1% penicillin-streptomycin (Thermo-Fisher Scientific, Waltham, MA, USA).

**Chromatographic purification:** HeLa S3 cells (5 × 10^10^ cells.) were obtained as a pellet from the National Cell Culture Center (Minneapolis, MN, USA) and nuclear extracts were prepared as described by Dignam *et al.* (Dignam et al., 1983). Nuclear extracts were dialyzed against buffer A containing 20 mM HEPES-NaOH (pH 7.6), 0.1 mM EDTA, 1 mM DTT, 10% (v/v) glycerol, 1 mM benzamidine, 0.25 mM PMSF, and 2 µg/mL aprotinin. The supernatant after centrifugation at 40,000 rpm for 30 min in a Ti-45 rotor was applied to a phosphocellulose column (10 mg protein/mL packed column bed volume) that was equilibrated in buffer A containing 0.1 M KCl, and was eluted stepwise with buffer A containing 0.3 M, 0.6 M, and 1.0 M KCl. Fractions (1/5 column volume) were collected. Enzymatically active fractions from the column eluted with 0.3 M KCl were subjected using the TSK DEAE-5PW column (7.5×75 mm; Toso-Haas Laboratory Service, Montgomeryville, PA, USA) that was pre-equilibrated in Buffer B (20 mM Hepes-NaOH (pH 7.9), 0.5 mM EDTA, 1 mM dithiothreitol (DTT), and 10% (v/v) glycerol) containing 0.05 M KCl at a 1 mL/min flow rate. Then the column was eluted at 1 mL/min with a 30 mL linear gradient from 0.05 M to 0.6 M KCl in Buffer B; 1.0 mL fractions were collected. Next, enzymatically active fractions from the DEAE-5PW column were applied to the TSK SP-5PW column (5×5 mm, Toso-Haas) that was pre-equilibrated in Buffer B containing 0.1 M KCl. The column was eluted at 0.5 mL/min with a linear salt gradient from 0.05 M to 0.6 M KCl. Then 0.5 mL fractions were collected. Finally, enzymatically active fractions from the SP-5PW column were applied to 10-40 % Glycerol gradient gel, after centrifugation at 55,000 rpm for 6 hours in a Ti-45 rotor, 100 µL fractions were collected. In the second time purification, enzymatically active fractions from the P11 column eluted with 0.3 M KCl were subjected using the TSK DEAE-5PW column (7.5×600 mm; Toso-Haas Laboratory Service, Montgomeryville, PA, USA) that was pre-equilibrated in Buffer B (40 mM Hepes-NaOH (pH 7.9), 0.5 mM EDTA, 1 mM dithiothreitol (DTT), and 10% (v/v) glycerol) containing 0.4 M KCl. Fractions were eluted at a 0.5 mL/min flow rate and collected at 0.5 ml size.

H2A-H2A.Z replacement activity of all fractions from each chromatography was measured by an *in vitro* H2A.Z deposition assay. Each experiment was performed 2 times.

**Preparation of histone octamers and mononucleosomes/polynucleosomes**

Recombinant human histone octamers and mononucleosomes (216 bp DNA fragment) were prepared as previously described (Cai et al., 2006). Then assembled nucleosomes were immobilized on Dynabeads M-280 Streptavidin (11206D; Invitrogen, Carlsbad, CA, USA).

***In vitro* H2A.Z-exchange assay**

A mixture of *E.coli* expressed/purified histone H2A or H2A.Z, purified endogenous DNA-PKcs-complex, ATP (100µM), and kinase buffer containing 50mM Tris-HCl (pH 7.5), 100mM KCl, 50mM MgCl2, 1mM Na3CO4, 1mM DTT, 5% glycerol were incubated at 25℃ for 1 hour. Then the reaction mixture was subjected to SDS-PAGE, and phosphor-modified H2A/H2A.Z was analyzed by western blot with phosphor-Ser/Thr-specific antibody (Cai et al., 2006).

***In vitro* phosphorylation assay**

Histone H2A-containing reconstituted nucleosomes, Flag-H2A.Z-H2B dimers, purified DNA-PKcs-containing component were used in the *in vitro* histone variant H2A.Z-exchange assay (Yang et al., 2012).

**Short hairpin RNA knockdown**

The pLVX-shRNA plasmids were purchased from Hanheng (Shanghai, China). pLVX-shRNA plasmids were transiently transfected using polyethylenimine (Cat No. 23966; Polysciences, Warrington, PA, USA) according to the manufacturer’s recommendation. The sequences targeting CAP-H2 and DNA-PKcs were as follows: shDNA-PKcs-1: 5’-CCAGAGATTTCGGTTTGCTTGTATT-3’, shDNA-PKcs-2: 5’-CAGACACTGGTGACGACCGTGTTTA-3’; shCAP-H2-1: 5’-GACGAAGGCAAGACCACAATG-3’, and shCAP-H2-2: 5’-CGGACTAACGTGGATCTCAAGAATG-3’.

**Mass spectrometry**

Anti-Flag purified DNA-PKcs-associated proteins, and multi-step chromatographic purified proteins were digested by trypsin, and the peptide sequences were analyzed by multi-dimensional protein identification technology (MudPIT) (Applied Protein Technology, Shanghai, China).

**Immunofluorescence staining**

Immunofluorescence staining was done as described previously [23]. Cells were incubated with DNA-PKcs (1 : 400) and H2A.Z (1 : 2000) primary antibodies at 37 °C for 1 h, and then stained with FITC-conjugated or TRITC-conjugated secondary antibodies (rabbit/green: 1 : 300, ZF-0313; mouse/red: 1:300, ZF-0311). Cell nuclei were stained with DAPI containing Vectashield (H-1200; Vector Laboratories, Inc., Burlingame, CA, USA). Fluorescence images were observed with the Olympus BX40F Microscope (Olympus Co., Tokyo, Japan).

**Chromatin immunoprecipitation assay (ChIP)**

Stably expressing *Asi*SI-ER-U2OS cells were cultured and grown to 80–90% confluence in a 10-cm plate. Cells were then harvested for ChIP assays at 0 h, 10 min, 30 min, 1 h and 2 h after 4-OHT treatment. ChIP experiment was performed with antibody directed against H2A.Z according to a standard protocol. ChIP DNA was amplified with qPCR. Each experiment was performed 2–3 times. Antibodies and IgG-ChIP signals were normalized to total input. qPCR primers located in close proximity to (3.7 kb) and distal (2 Mb) from the AsiSI site on chromosome 22 at position 19180307 (Lacovoni et al., 2010) were: proximal site (3.7kb), forward: 5’-CCCATCTCAACCTCCACACT-3’; reverse: 5’-CTTGTCCAGATTCGCTGTGA-3’; distal site (2 Mb): forward: 5’-CCTTCTTTCCCAGTGGTTCA-3’; forward: 5’-GTGGTCTGACCCAGAGTGGT-3’ (Qi et al., 2015).

**References**

Dignam JD, Lebovitz RM, Roeder RG (1983) Accurate transcription initiation by RNA polymerase II in a soluble extract from isolated mammalian nuclei. Nucleic Acids Res 11(5): 1475-1489.

Cai Y, Jin J, Gottschalk AJ, Yao T, Conaway JW, Conaway RC (2006) Purification and assay of the human INO80 and SRCAP chromatin remodeling complexes. Methods 40(4):312-317.

Qi W, Wang ., Chen H, Wang X, Xiao T, Boldogh I, Ba X, Han L, Zeng X (2015) BRG1 promotes the repair of DNA double-strand breaks by facilitating the replacement of RPA with RAD51. J Cell Sci 128: 317-330.

Yang W, Xia Y, Hawke D, Li X, Liang J, Xing D, Aldape K, Hunter T, Alfred Yung WK, Lu Z (2012) PKM2 phosphorylates histone H3 and promotes gene transcription and tumorigenesis. Cell 150:685-696.

**Figures**


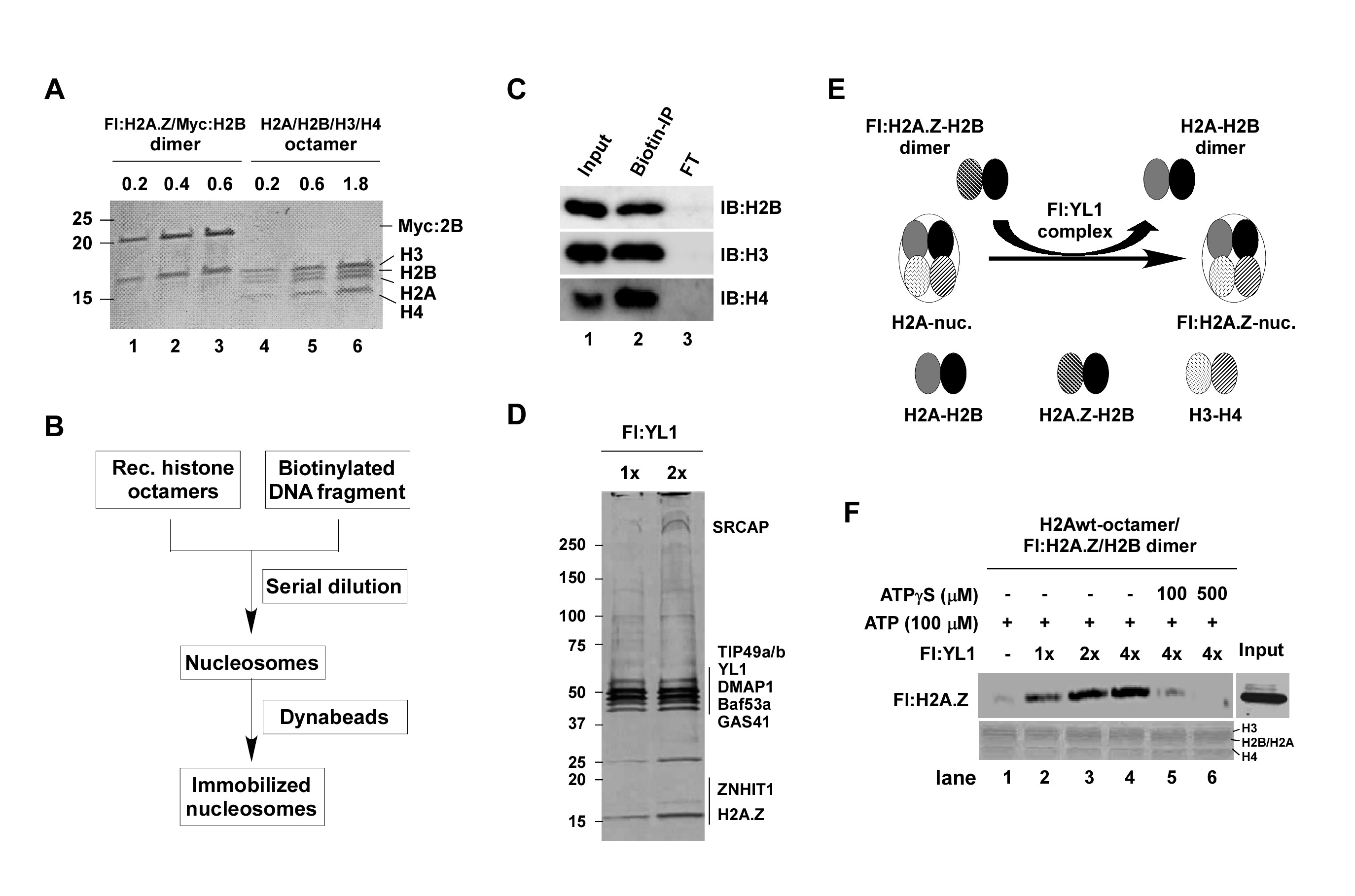


Figure S1. *In vitro* H2A.Z-H2A replacement assay was established using reconstituted nucleosomes and dimers. (A) Coomassie Brilliant Blue (CBB) stained gel. Histone Flag-H2A.Z-H2B dimers and H2A/H2B/H3/H4 octamers were reconstituted using E.coli expressing and purified histones. Histone proteins were analyzed by SDS-PAGE and stained by CBB. (B) Schema of the reconstitution of immobilized nucleosomes. (C) Verification of nucleosomal arrays. (D) Silver stained gel. Anti-Flag eluates from stably expressing Flag-YL1 293FRT cells were subjected to 4%–15% gradient SDS-PAGE, and Flag-YL1 and its associated proteins were visualized by silver staining. (E) *In vitro* H2A.Z replacement enzyme activity detection diagram. (F) Establishment of H2A.Z deposition enzyme activity. *In vitro* assay was conducted by incubating immobilized nucleosomes, H2A.Z-H2B dimers, Flag-YL1, and ATP. H2A.Z incorporated into nucleosomes was detected by western blotting with anti-Flag antibody (upper panel). Histone octamers in each reaction were visualized by CBB staining (lower panel).


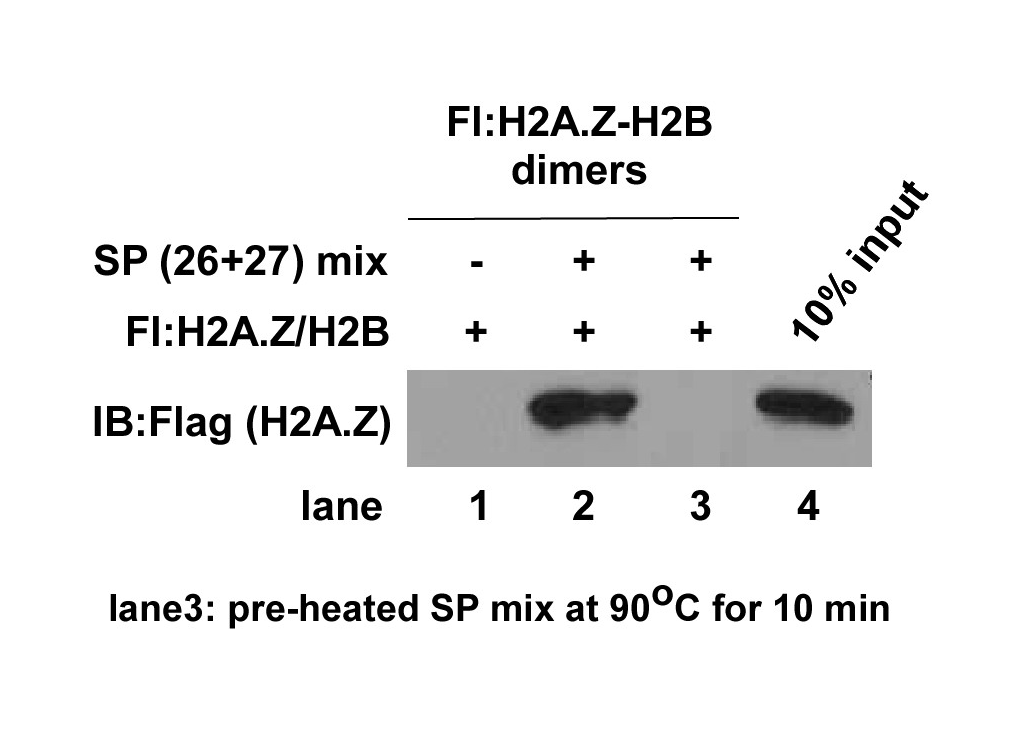


Figure S2. Enzyme activity was related to protein components. A mixture of SP-5PW fractions 26–27 was heated at 90°C for 10 min before enzyme activity was detected.

| **Supplemental Table 1: Proteins from main fractions of 10-40% Glycerol gradient identified by MudPIT** | | |
| --- | --- | --- |
| On the rows showing the **locus names** are reported the sequence coverage (percentage of the protein sequence covered by identified peptides) measured for a particular analysis. Numbers in rows showing individual peptides are cross-correlation scores. | | |
| **Locus names** | **10-40% Glycerol gradient (sequence coverage%)** | **Description** |
| **gi\|13654237\|ref\|NP_008835.5** | **42.50** | **DNA-dependent protein kinase catalytic subunit isoform 1** |
| R.LQETLSAADR.C +2 |  |  |
| R.CGAALAGHQLIR.G +2 |  |  |
| R.DFGLLVFVR.K +2 |  |  |
| K.FLCIFLEK.M +1 |  |  |
| K.IPALDLLIK.L +2 |  |  |
| K.FYGELALKK.K +2 |  |  |
| K.IPDTVLEK.V +2 |  |  |
| R.AFLGELK.T +1 |  |  |
| K.TQMTSAVREPK.L +2 |  |  |
| K.LPVLAGCLK.G +2 |  |  |
| K.GLSSLLCNFTK.S +2 |  |  |
| R.EIFNFVLK.A +1 |  |  |
| K.AIRPQIDLKR.Y +2 |  |  |
| K.RYAVPSAGLR.L +2 |  |  |
| K.AALSALESFLK.Q +2 |  |  |
| K.LQYFMEQFYGIIR.N +2 |  |  |
| K.QMFLTQTDTGDDR.V +2 |  |  |
| K.DYVDLFR.H +2 |  |  |
| D.PAANLHPAKPK.D +1 |  |  |
| K.DFSAFINLVEFCR.E +2 |  |  |
| R.LPLISGFYK.L +2 |  |  |
| K.YFEGVSPK.S +1 |  |  |
| K.YSCFALFVK.F +2 |  |  |
| K.DILPCLDGYLK.T +2 |  |  |
| K.NNWEVSALSR.A +2 |  |  |
| K.NLSSNEAISLEEIR.I +2 |  |  |
| R.VVQMLGSLGGQINK.N +2 |  |  |
| K.SYVAWDREK.R +2 |  |  |
| R.LSFAVPFR.E +1 |  |  |
| R.EMKPVIFLDVFLPR.V +2 |  |  |
| K.PVIFLDVFLPR.V +2 |  |  |
| R.VTELALTASDRQTK.V +2 |  |  |
| K.RTFPVLLR.L +2 |  |  |
| R.LACDVDQVTR.Q +2 |  |  |
| R.QLYEPLVMQLIHWFTNNK.K +2 |  |  |
| R.LYSLALHPNAFK.R +2 |  |  |
| Y.SLALHPNAFK.R +2 |  |  |
| R.LGASLAFNNIYR.E +2 |  |  |
| K.SLGTIQQCCDAIDHLCR.I +3 |  |  |
| K.WLLAHCGRPQTECR.H +2 |  |  |
| K.SIELFYKFVPLLPGNR.S +2 |  |  |
| K.FVPLLPGNRSPNLWLK.D +2 |  |  |
| N.RSPNLWLKDVLK.E +2 |  |  |
| F.EGGGCGQPSGILAQPTLLYLR.G +2 |  |  |
| R.TVGALQVLGTEAQSSLLK.A +3 |  |  |
| K.CFGTGAAGNR.T +2 |  |  |
| K.DLCNTHLMR.V +2 |  |  |
| K.DILETHLR.E +2 |  |  |
| K.ITAQSIEELCAVNLYGPDAQVDR.S +2 |  |  |
| R.LAAVVSACK.Q +2 |  |  |
| R.QCLPSLDLSCK.Q +2 |  |  |
| K.QLASGLLELAFAFGGLCER.L +2 |  |  |
| K.NLDLAVLELMQSSVDNTK.M +3 |  |  |
| K.LATTILQHWK.K +2 |  |  |
| K.MAVLALLAK.I +2 |  |  |
| R.FNNYVDCMK.K +2 |  |  |
| R.EQQHVMEELFQSSFR.R +2 |  |  |
| R.EFFSTIVVDAIDVLK.S +2 |  |  |
| K.LNESTFDTQITKK.M +2 |  |  |
| K.ILDVMYSR.L +2 |  |  |
| R.LPKDDVHAK.E +2 |  |  |
| K.INQVFHGSCITEGNELTK.T +3 |  |  |
| K.FYQGFLFSEKPEK.N +2 |  |  |
| K.NLLIFENLIDLKR.R +2 |  |  |
| R.HECMAPLTALVK.H +2 |  |  |
| R.SLGPPQGEEDSVPR.D +2 |  |  |
| K.LGNPIVPLNIR.L +2 |  |  |
| K.LVINTEEVFRPYAK.H +2 |  |  |
| K.EVYAAAAEVLGLILR.Y +2 |  |  |
| K.NILEESLCELVAK.Q +2 |  |  |
| K.SKDFVQVMR.H +2 |  |  |
| K.VCLDIIYK.M +2 |  |  |
| R.ELLNPVVEFVSHPSTTCR.E +2 |  |  |
| K.DVLIQGLIDENPGLQLIIR.N +3 |  |  |
| R.LLALNSLYSPK.I +2 |  |  |
| R.STVLTPMFVETQASQGTLQTR.T +3 |  |  |
| R.WPVAGQIR.A +2 |  |  |
| R.ATQQQHDFTLTQTADGR.S +3 |  |  |
| R.SSFDWLTGSSTDPLVDHTSPSSDSLLFAHK.R +3 |  |  |
| D.PLVDHTSPSSDSLLFAHK.R +2 |  |  |
| K.SVGPDFGKK.R +2 |  |  |
| K.RLGLPGDEVDNK.V +2 |  |  |
| K.MKQDAQVVLYR.S +2 |  |  |
| K.QDAQVVLYR.S +2 |  |  |
| R.SYRHGDLPDIQIK.H +2 |  |  |
| R.HGDLPDIQIK.H +2 |  |  |
| K.HSSLITPLQAVAQRDPIIAK.Q +3 |  |  |
| S.SLITPLQAVAQRDPIIAK.Q +2 |  |  |
| K.QLFSSLFSGILK.E +2 |  |  |
| D.PAAVSAGCLASLQQPVGIR.L +2 |  |  |
| C.LASLQQPVGIR.L +2 |  |  |
| R.LLPAELPAK.R +2 |  |  |
| K.ARLPPDVLR.W +2 |  |  |
| R.GIFTSEIGTK.Q +2 |  |  |
| K.QITQSALLAEAR.S +2 |  |  |
| K.QDWVDGEPTEAEK.D +2 |  |  |
| K.DFWELASLDCYNHLAEWK.S +2 |  |  |
| K.SLEYCSTASIDSENPPDLNK.I +2 |  |  |
| K.LLLQGEADQSLLTFIDK.A +2 |  |  |
| Y.SSIDVLLHQSR.L +2 |  |  |
| K.LQSVQALTEIQEFISFISK.Q +3 |  |  |
| K.QGNLSSQVPLK.R +2 |  |  |
| R.LLNTWTNRYPDAK.M +2 |  |  |
| R.CFFLSKIEEK.L +2 |  |  |
| R.MEVQEQEEDISSLIR.S +2 |  |  |
| R.KQNNFSLAMK.L +2 |  |  |
| R.DDWLVSWVQSYCR.L +2 |  |  |
| R.SQGCSEQVLTVLK.T +2 |  |  |
| K.TVSLLDENNVSSYLSK.N +2 |  |  |
| R.DQNILLGTTYR.I +2 |  |  |
| R.IIANALSSEPACLAEIEEDK.A +2 |  |  |
| N.ALSSEPACLAEIEEDK.A +2 |  |  |
| R.ILELSGSSSEDSEK.V +2 |  |  |
| K.VIAGLYQR.A +2 |  |  |
| Y.MTLADFCDQQLR.K +2 |  |  |
| R.KEEENASVIDSAELQAYPALVVEK.M +3 |  |  |
| R.LLQIIERYPEETLSLMTK.E +2 |  |  |
| R.YPEETLSLMTK.E +2 |  |  |
| F.IISSESYSFK.D +2 |  |  |
| K.LDQGGVIQDFINALDQLSNPELLFK.D +2 |  |  |
| K.DWSNDVR.A +1 |  |  |
| R.MYAALGDPK.A +2 |  |  |
| R.KFIQTFGKEFDK.H +2 |  |  |
| K.FIQTFGKEFDK.H +2 |  |  |
| K.VEFLRNELEIPGQYDGR.G +2 |  |  |
| R.NELEIPGQYDGR.G +2 |  |  |
| R.GKPLPEYHVR.I +1 |  |  |
| R.GHDEREHPFLVK.G +2 |  |  |
| R.VEQLFQVMNGILAQDSACSQR.A +3 |  |  |
| R.TYSVVPMTSR.L +2 |  |  |
| R.LGLIEWLENTVTLK.D +2 |  |  |
| K.DLLLNTMSQEEK.A +2 |  |  |
| K.AAYLSDPRAPPCEYKDWLTK.M +3 |  |  |
| K.GANRTETVTSFR.K +2 |  |  |
| R.MSTSPEAFLALR.S +2 |  |  |
| R.SDPGLLTNTMDVFVK.E +2 |  |  |
| D.PGLLTNTMDVFVK.E +2 |  |  |
| K.KGGSWIQEINVAEK.N +3 |  |  |
| K.LAGANPAVITCDELLLGHEK.A +3 |  |  |
| K.APAFRDYVAVAR.G +2 |  |  |
| R.AQEPESGLSEETQVK.C +2 |  |  |
|  |  |  |
| **gi\|41149476\|ref\|XP_166201.2\|** | **17.60** | **Structural maintenance of chromosomes 2-like 1 (SMC2)** |
| R.TFAAQSLVQLLSK.L +2 |  |  |
| K.SALQVLVSILK.H +2 |  |  |
| K.ALEFLDQLLLQNIR.H +2 |  |  |
| K.LNGFQWSLEVISSAVDALQR.L +3 |  |  |
| K.YIFTLGDIAQLCPAR.V +2 |  |  |
| R.AHAIITLGK.L +1 |  |  |
| R.NFIENIIPIIISLK.T +2 |  |  |
| K.KYQEQLVQEQELAK.H +2 |  |  |
| R.ASAGHVAVSSPTPETGPLQR.L +2 |  |  |
| R.SLGVLPFTLNSGSPEK.T +2 |  |  |
| R.SQGNDILCLSLPDKPPPQPQQWNVR.S +2 |  |  |
|  |  |  |
| **gi\|41149476\|ref\|XP_166201.2** | **12.30** | **KIAA0056 protein (hCAP-D2)** |
| R.TFAAQSLVQLLSK.L +2 |  |  |
| K.SALQVLVSILK.H +2 |  |  |
| R.GVVPVVMDCESTVQEK.A +2 |  |  |
| K.ALEFLDQLLLQNIR.H +2 |  |  |
| K.LNGFQWSLEVISSAVDALQR.L +3 |  |  |
| K.YIFTLGDIAQLCPAR.V +2 |  |  |
| R.AHAIITLGK.L +1 |  |  |
| R.NFIENIIPIIISLK.T +2 |  |  |
| K.KYQEQLVQEQELAK.H +2 |  |  |
| R.ASAGHVAVSSPTPETGPLQR.L +2 |  |  |
| R.SLGVLPFTLNSGSPEK.T +2 |  |  |
| R.SQGNDILCLSLPDKPPPQPQQWNVR.S +3 |  |  |
|  |  |  |
| **gi\|21361252\|ref\|NP_005487.2\|** | **10.90** | **Structural maintenance of chromosomes 4-like 1 (SMC4)** |
| R.TESPATAAETASEELDNR.S +2 |  |  |
| K.NIAIEFLTLENEIFR.K +2 |  |  |
| K.FTQLDLEDVQVR.E +2 |  |  |
| K.LTQEETNFK.S +2 |  |  |
| K.SLVHDLFQK.V +2 |  |  |
| R.QNIGVATFIGLDK.M +2 |  |  |
| K.KMTEIQTPENTPR.L +2 |  |  |
| K.K.VIQENEHALQK.D +2 |  |  |
| K.NPDSITNQIALLEAR.C +2 |  |  |
|  |  |  |
| **gi\|11024714\|ref\|NP_061828.1\|** | **7.00** | **Ubiquitin B precursor; polyubiquitin B** |
| K.TITLEVEPSDTIENVK.A +2 |  |  |
|  |  |  |
| **gi\|41146985\|ref\|XP_371762.1\|** | **6.70** | **Similar to Proteasome activator complex subunit 2** |
| V.AIQEKVLERVNAVKIK.V +2 |  |  |
|  |  |  |
| **gi\|7549803\|ref\|NP_054644.1\|** | **6.60** | **Deiodinase, iodothyronine, type II** |
| K.IAEGATCHLLDFASPERP.L +2 |  |  |
|  |  |  |
| **gi\|40255237\|ref\|NP_060230.4\|** | **6.30** | **Hypothetical protein FLJ20311 (IhCAP-G)** |
| K.RETFVQAVSK.E +2 |  |  |
| K.ELVGEFLQFVQLDK.E +2 |  |  |
| R.EVLSYFHHQK.K +2 |  |  |
| K.FASVLPEYLK.V +2 |  |  |
| K.NRECLLSAPR.K +2 |  |  |
| R.GVLSTLIAGPVVEISHQLR.K +2 |  |  |
|  |  |  |
| **gi\|23097285\|ref\|NP_689512.1\|** | **6.30** | **Hypothetical protein MGC18000 (hCAP-H2)** |
| K.NNNPLYSR.Q +2 |  |  |
| R.MNTCVPHPR.G +2 |  |  |
| K.ETPDPWQSLDPFDSLESK.P +2 |  |  |
|  |  |  |
| **gi\|14327896\|ref\|NP_114172.1\|** | **6.20** | **G2/mitotic-specific cyclin B1** |
| MALRVTRNSKINAEN.K +2 |  |  |
| P.EPEPEPEPEPVK.E +2 |  |  |
|  |  |  |
| **gi\|17017976\|ref\|NP_003387.1\|** | **5.90** | **Wingless-type MMTV integration site family, member 9B precursor** |
| A.AAPAQGGAHLKQCDLLKLSRR.Q +2 |  |  |
|  |  |  |
| **gi\|23312371\|ref\|NP_690593.1\|** | **4.90** | **Tumor necrosis factor receptor superfamily, member 5 isoform 2 precursor;** |
| R.LPLQCVLWGC.L +2 |  |  |
|  |  |  |
| **gi\|21361794\|ref\|NP_060918.2\|** | **4.70** | **TIP120 protein** |
| K.TVIGELPPASSGSALAANVCK.K +2 |  |  |
| K.ALTLIAGSPLK.I +2 |  |  |
| K.EGPAVVGQFIQDVK.N +2 |  |  |
| K.LTLIDPETLLPR.L +2 |  |  |
|  |  |  |
| **gi\|41151351\|ref\|XP_373940.1\|** | **4.40** | **Hypothetical protein XP_379027** |
| M.DSVSPSGSR.W +1 |  |  |
|  |  |  |
| **gi\|4885049\|ref\|NP_005150.1\|** | **4.30** | **Actin, alpha** |
| R.SYELPDGQVITIGNER.F +2 |  |  |
|  |  |  |
| **gi\|32307161\|ref\|NP_003583.2\|** | **3.60** | **Cullin 1** |
| K.QIGLDQIWDDLR.A +2 |  |  |
| K.SPELLAR.Y +1 |  |  |
| K.LLIQAAIVR.I +2 |  |  |
|  |  |  |
| **gi\|4758294\|ref\|NP_004437.1\|** | **3.30** | **Glutamyl-prolyl tRNA synthetase** |
| K.INEAVECLLSLK.A +2 |  |  |
| K.NQGGGLSSSGAGEGQGPK.K +2 |  |  |
| K.SLCIPFKPLCELQPGAK.C +3 |  |  |
|  |  |  |
| **gi\|42656354\|ref\|XP_376074.1\|** | **2.50** | **Hypothetical protein XP_376074** |
| I.YGETERK.R +1 |  |  |
|  |  |  |
| **gi\|4827008\|ref\|NP_005064.1\|** | **2.10** | **Solute carrier family 15** |
| K.VFEDISANTVNMALQ.I +2 |  |  |
|  |  |  |
| **gi\|4502567\|ref\|NP_003679.1\|** | **2.00** | **Calcium/calmodulin-dependent serine protein kinase** |
| T.VEQLQKMLREMRGSITFK.I +2 |  |  |
|  |  |  |
| **gi\|28875784\|ref\|NP_079018.1\|** | **1.80** | **Hypothetical protein FLJ13063** |
| Q.LAAAAGEALGGEK.D +2 |  |  |
|  |  |  |
| **gi\|41194247\|ref\|XP_029323.3\|** | **1.80** | **Similar to KRT8 protein** |
| K.NKYEDEINK.H +2 |  |  |
|  |  |  |
| **gi\|29337159\|sp\|O43513\|** |  | **FLJ42925 protein** |
| V.QPLGKFR.A +1 |  |  |
|  |  |  |
| **gi\|14043022\|ref\|NP_004981.2\|** | **1.60** | **Methionine-tRNA synthetase** |
| K.QLAVAEGKPPEAPK.G +2 |  |  |
|  |  |  |
| **gi\|38569489\|ref\|NP_942568.1\|** | **1.40** | **KIAA0676 protein** |
| R.GGGLTGLLVGTLDVVLD.S +2 |  |  |
|  |  |  |
| **gi\|4826960\|ref\|NP_005042.1\|** | **1.40** | **Glutaminyl-tRNA synthetase** |
| K.ILQLVATGAVR.D +2 |  |  |
|  |  |  |
| **gi\|4502745\|ref\|NP_001251.1\|** | **1.30** | **PR domain containing 10 isoform 1** |
| gi\|41349458\|ref\|NP_064613.2 +2 |  |  |
|  |  |  |
| **gi\|24496789\|ref\|NP_064502.8\|** | **1.20** | **Leucyl-tRNA synthetase** |
| K.STGNFLTLTQAIDK.F +2 |  |  |
|  |  |  |
| **gi\|32469501\|ref\|NP_862824.1\|** | **1.20** | **Transmembrane channel-like 3** |
| K.XXXXXXXXXXXXX.X +2 |  |  |
|  |  |  |
| **gi\|13324688\|ref\|NP_060587.8\|** | **1.00** | **WD repeat domain 11** |
| I.GQSAIAGEEHPR.G +2 |  |  |
|  |  |  |
| **gi\|42656010\|ref\|XP_057107.5\|** | **0.90** | **KIAA1937 protein** |
|  |  |  |
| **gi\|41393563\|ref\|NP_055889.2\|** | **0.80** | **Kinesin family member 1B isoform b** |
| S.SVAMTRSGLSLEELR.I +2 |  |  |
|  |  |  |
| **gi\|4504555\|ref\|NP_002152.1\|** | **0.80** | **Isoleucine-tRNA synthetase** |
| R.LYLINSPVVR.A +2 |  |  |
|  |  |  |
| **gi\|41149741\|ref\|XP_058513.7\|** | **0.80** | **Similar to RIKEN cDNA 4921513O20** |
| R.MYWRQGIYLN.W +2 |  |  |
|  |  |  |
| **gi\|32698688\|ref\|NP_009105.1\|** | **0.40** | **Serine/threonine kinase 21** |
| R.YNENLSKY.C +1 |  |  |
|  |  |  |
| **gi\|24415404\|ref\|NP_055426.1\|** | **0.20** | **MDN1, midasin homolog** |
| F.AWRDGPLLAALK.A +2 |  |  |
|  |  |  |
| **Criteria sets:** |  |  |
| Minimum +1 XCorr |  |  |
| Minimum +2 XCorr |  |  |
| Minimum +3 XCorr | \| 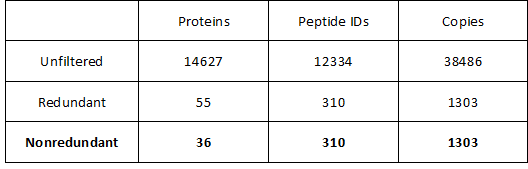 \| \| --- \| |  |
| Minimum DeltCN |  |  |
| Minimum charge state |  |  |
| Maximum charge state |  |  |
| Tryptic status requirement |  |  |
| Multiple, ambiguous IDs allowed |  |  |
| Minimum sequence length |  |  |
| Purge duplicate peptides by protein |  |  |
| Include only loci with unique peptide |  |  |
| Remove subset proteins |  |  |
| Exclude protein names matching |  |  |
| Exclude protein descriptions matching |  |  |
| Include only protein descriptions matching |  |  |
| Minimum redundancy for low coverage loci |  |  |
| Minimum peptides per locus |  |  |
|  |  |  |
